# Quantitative Dynamic Contrast-Enhanced Magnetic Resonance Imaging and Positron Emission Tomography (PET) for Distinguishing Metastatic Lymph Nodes from Nonmetastatic Among Patients with Rectal Cancer: A Systematic Review and Meta-Analysis

**DOI:** 10.1055/s-0044-1788794

**Published:** 2024-08-06

**Authors:** Seyedeh Nooshin Miratashi Yazdi, Sahand Adib Moradi, Seyedeh Sahel Rasoulighasemlouei, Fatemeh Parouei, Mohamad Ghazanfari Hashemi

**Affiliations:** 1 Department of Radiology, Tehran University of Medical Sciences, Tehran, Iran; 2 Iranian Center of Neurological Research, Neuroscience Institute, Tehran University of Medical Sciences, Tehran, Iran; 3 Advanced Diagnostic and Interventional Radiology Research Center (ADIR), Tehran University of Medical Sciences, Tehran, Iran; 4 Department of Gastroenterology and Hepatology, Erasmus University Medical Center, Rotterdam, The Netherlands; 5 Cancer Institute, Tehran University of Medical Sciences, Tehran, Iran

**Keywords:** QDCE-MRI, quantitative dynamic contrast-enhanced magnetic resonance imaging, positron emission tomography, metastatic lymph node, rectal cancer

## Abstract

**Objective** The objective of this research was to assess the proficiency of quantitative dynamic contrast-enhanced magnetic resonance imaging (QDCE-MRI) and positron emission tomography (PET) imaging in distinguishing between metastatic and nonmetastatic lymph nodes in cases of rectal carcinoma.

**Method** This meta-analysis was conducted following the Preferred Reporting Items for Systematic Reviews and Meta-Analyses standards. Two independent reviewers systematically searched databases including PubMed, Embase, Web of Science, and the Cochrane Library. The research took place in July 2022, with no restriction on the initial date of publication. For the analysis, we utilized Stata software (version 16.0), Review Manager (version 5.3), and the Open Meta-Analyst computational tool.

**Results** A total of 19 studies consisting of 1,451 patients were included in the current meta-analysis. The differences between metastatic and nonmetastatic lymph node parameters were significant by using short axis and Ktrans (6.9 ± 4 vs. 5.4 ± 0.5, 0.22 ± 0.1 vs. 0.14 ± 0.1, respectively). Contrast-enhanced MRI (CE-MRI) showed 73% sensitivity, 71% specificity, and 79% accuracy in detecting metastatic lymph nodes among rectal cancer patients based on six included studies ( *n* = 530). The overall sensitivity, specificity, and accuracy of QDCE-MRI using Ktrans was calculated to be 80, 79, and 80%, respectively. Furthermore, PET-computed tomography (CT) showed a sensitivity of 80%, specificity of 91%, and accuracy of 86% in distinguishing metastatic lymph nodes. Quality utility analysis showed that using CE-MRI, QDCE-MRI, and PET-CT would increase the posttest probability to 69, 73, and 85%, respectively.

**Conclusion** QDCE-MRI demonstrates a commendable sensitivity and specificity, but slightly overshadowed by the higher specificity of PET-CT at 91%, despite comparable sensitivities. However, the heterogeneity in PET-CT sensitivity across studies and its high specificity indicate variability that can influence clinical decision-making. Thus, combining these imaging techniques and perhaps newer methods like PET/MRI could enhance diagnostic accuracy, reduce variability, and improve patient management strategies in rectal cancer.

## Introduction

Globally, rectal cancer (RC) ranks as a primary contributor to cancer-related fatalities. As per GLOBOCAN 2021 statistics, RC is positioned 8th in global incidence (3.9%) and mortality (3.2%) among all cancer types, affecting 732,210 individuals annually. 1 Even with advancements in early detection and intervention, the morbidity and mortality indices remain elevated, showing a 5-year survival rate of 64.7%. 2 Lymph node (LN) involvement serves as a crucial prognostic determinant in RC, 3 and as a result, neoadjuvant treatment prior to surgery is prescribed to minimize local recurrence rates. 4 When assessing LN involvement in RC using morphology-centric magnetic resonance imaging (MRI), sensitivity and specificity stand at 66 and 76%, respectively, based on a meta-analysis. 5 This underscores the need for refining the discrimination accuracy, possibly because morphological MRI seldom yields substantive functional tissue data.

Innovations in functional MRI methodologies, such as conventional and dynamic contrast-enhanced MRI (DCE-MRI)—offering insights into tissue microenvironments—have been shown effective in distinguishing benign from malignant formations across multiple organs including the pancreas, 6 7 breast, 8 and LNs. 9 10 It is widely acknowledged that angiogenesis and vascular traits are pivotal for tumor growth and its invasive nature. A study by Lamer et al 11 indicated that LNs affected by squamous cell carcinoma from the head and neck region possessed more vasculature than their nonmetastatic counterparts. Thus, by its very nature, DCE-MRI, with its capacity to delineate tissue vascularity and perfusion, might be more adept than traditional morphological MRI in discerning malignant LNs from benign ones.

When it comes to quantitative DCE-MRI (QDCE-MRI) analysis, which is grounded in the pharmacokinetic model, it quantifies perfusion-related metrics by gauging the tissue concentration of contrast mediums. This offers a perceived edge over the semiquantitative DCE-MRI approach in measuring tissue microcirculation. 12

On the other hand, the application of positron emission tomography (PET), including both PET/MRI and PET/computed tomography (CT), is a common practice in the management of RC, particularly for initial staging, reassessment, and identifying recurrence. 13 While this method is highly specific in identifying LN metastases, its sensitivity leaves room for improvement. The presence of increased [18F]fludeoxyglucose (FDG) absorption in LNs on PET scans is a significant indicator of LN metastases. 14 However, not every instance of elevated [18F]FDG levels in LNs can be attributed to cancer metastases. Misinterpretations can occur, with normal structures like venous plexuses or LNs inflamed due to other causes being mistaken for metastatic spread. Therefore, relying solely on [18F]FDG uptake in LNs as an indicator for metastases is flawed, underscoring the need for more accurate diagnostic approaches. 14

To date, there is a lack of existing research on the efficacy of QDCE-MRI, PET, or combinational imaging in differentiating metastatic LNs from their nonmetastatic counterparts in the context of RC. This research aims to examine the proficiency of QDCE-MRI and PET in distinguishing metastatic LNs from nonmetastatic ones in rectal malignancies.

## Methodology

This meta-analysis was structured and presented in compliance with the Preferred Reporting Items for Systematic Reviews and Meta-Analyses guidelines. 15

### Search Strategy for Literature

The databases of PubMed, Embase, Web of Science, and Cochrane Library were meticulously probed by a pair of independent reviewers to pinpoint relevant studies. This search was executed in July 2022, unrestricted by the commencement date. Our exploration utilized keywords such as “magnetic resonance imaging,” “MRI,” “dynamic contrast-enhanced MRI,” “DCE-MRI,” “rectal carcinoma,” “metastasis,” “lymph node,” “Positron Emission Tomography,” “PET/CT,” “PET/MRI,” and “PET.” Both Medical Subject Headings terms and their diverse adaptations were employed. We limited our scope to English publications and manually scrutinized reference lists from pertinent articles to unearth additional fitting studies. Differences in opinions were deliberated until consensus was achieved.

### Criteria for Including Studies

Potential studies had their titles and summaries reviewed for suitability by two evaluators. Any divergence in opinions was deliberated upon until a common ground was found. Selected studies adhered to the subsequent benchmarks: (1) they were original investigative works; (2) they encompassed patients with RC validated via biopsy or histological analysis; and (3) they had undergone DCE-MRI and/or PET/CT and/or PET/MRI to evaluate LNs or presented adequate data to formulate the 2 × 2 contingency table for deducing sensitivity and specificity.

Exclusion criteria were: (1) summaries, editorial pieces, reviews, animal-based research, and conference discussions; (2) repetitive reports for an identical cohort (in such instances, the most comprehensive report was favored for inclusion); and (3) studies including less than 20 patients.

### Data Collection

From every pertinent study, we extracted crucial information like the principal author, year of publication, cohort size, details on MRI, data origins, and benchmark standards. For every qualifying study, the data on true positive, false positive (FP), false negative, and true negative were sourced, leading to the formation of a 2 × 2 contingency matrix.

### Assessment of Data Integrity

For evaluating the methodological integrity and potential biases of the incorporated studies, the Quality Assessment of Diagnostic Accuracy Studies (QUADAS-2) and Radiomics Quality Score (RQS) tools were employed. 16 17 The RQS criteria encompass: (1) capturing of images, (2) derivation of radiomics features, (3) data model development, (4) model validation, and (5) sharing of data. Every item from the RQS's 16 elements gets a score, resulting in an overall score varying between −8 (indicative of 0%) to 36 (indicative of 100%). 18

The QUADAS-2 tool integrates elements like (1) selection of patients, (2) the main test, (3) the gold standard, and (4) sequence and duration. A duo of independent evaluators undertook the quality evaluation, and disputes, if any, were settled by engaging a third evaluator for a unanimous decision. 19

### Analysis of Data

The statistical analysis for this meta-analysis utilized tools such as Stata software (version 16.0), Review Manager (version 5.3), and Open Meta-Analyst. Predictive precision was determined through pooled sensitivity, specificity, area under the curve (AUC), positive likelihood ratio (PLR), and negative likelihood ratio—all accompanied by 95% confidence intervals (CIs). The diagnostic precision was collectively represented using the summary receiver operating characteristic curve and AUC.

## Results

### Search Strategy for Literature

**Fig. 1:**
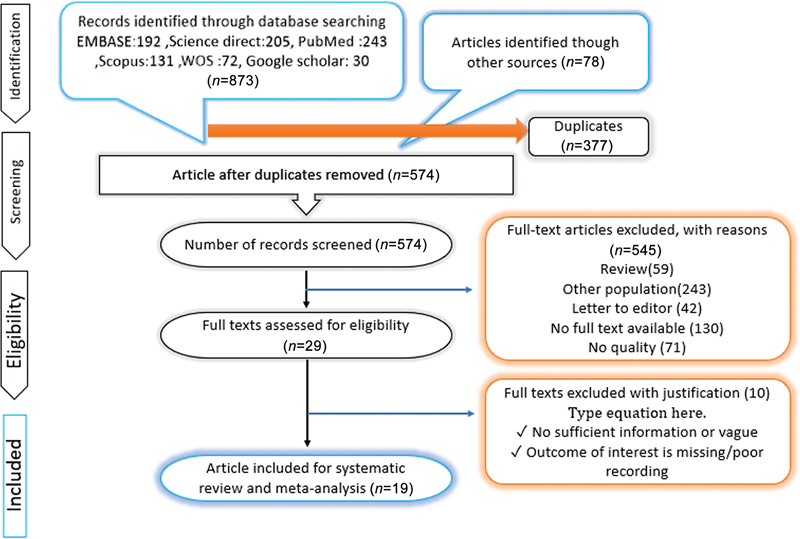
Preferred Reporting Items for Systematic Reviews and Meta-Analyses (PRISMA) diagram for the inclusion of studies.

Fig. 1 outlines the comprehensive literature search process. Following the previously detailed search methodology, we found 951 potential studies for consideration. With the removal of 377 redundant entries, 574 studies remained for assessment. Postpreliminary evaluation of titles and abstracts, 545 studies were discarded as they fell outside the inclusion parameters. Subsequent detailed scrutiny led to the exclusion of an additional 10 papers, leading to the selection of 19 studies for incorporation into the meta-analysis (as visualized in Fig. 1 ).

### Characteristics of the Included Studies

The characteristics of the included studies are summarized in Table 1 . Nineteen studies that were included in this meta-analysis had 1,451 patients. Six out of these studies were prospective and the rest had a retrospective design. Among 19 studies, 7 used contrast-enhanced MRI (CE-MRI), 4 used dynamic quantitative CE-MRI, 6 used PET/CT, and 2 used PET/MRI. The mean age of included patients was 62.3 ± 4 years ( Table 2 ).

**Table 1:** Comparison of the QDCE-MRI measurements among metastatic and nonmetastatic lymph nodes in patients with rectal cancer

Author/year	Number		Ktrans (min ^−1^ )		V _e_		Kep (min ^−1^ )	
	Metastatic	Nonmetastatic	Metastatic	Nonmetastatic	Metastatic	Nonmetastatic	Metastatic	Nonmetastatic
Yu/2016	35	44	0.483 ± 0.198	0.218 ± 0.116	0.399 ± 0.118	0.203 ± 0.096	1.263 ± 0.496	1.311 ± 0.794
Yang/2019	27	38	0.07 ± 0.03	0.10 ± 0.04	0.24 ± 0.15	0.28 ± 0.20	0.30 ± 0.12	0.32 ± 0.15
Yu/2017	40	23	0.562 ± 0.271	0.343 ± 0.151	0.358 ± 0.204	0.250 ± 0.078	1.659 ± 0.580	1.372 ± 0.339
Yeo/2015	7	18	0.101 ± 0.038	0.116 ± 0.044	0.373 ± 0.140	0.529 ± 0.177	0.443 ± 0.1	0.529 ± 0.17

Abbreviation: QDCE-MRI, quantitative dynamic contrast-enhanced magnetic resonance imaging.

**Table 2:** Characteristics of included studies

Author/year	Country	Design	Sample size (patients)	Sample size (lymph nodes)	Male/female	Mean age	Imaging method
Yu/2016 39	China	Prospective	59	82	27/32	53	DQCE-MRI
Yang/2019 40	China	Prospective	122	2,164	68/54	58.96	DQCE-MRI
Yu/2017 41	China	Retrospective	63	N/A	39/24	58.5	DQCE-MRI
Yeo/2015 42	South Korea	Prospective	46	31	34/12	62	DQCE-MRI
Doyon/2015 43	Germany	Retrospective	65	N/A	20/45	63	CE-MRI
Ogawa/2016 44	Japan	Retrospective	226	N/A	304/145	62.2	CE-MRI
Gröne/2018 45	Germany	Retrospective	60	N/A	39/21	64.6	CE-MRI
Kim/2018 46	South Korea	Retrospective	57	608	33/24	57	CE-MRI
Armbruster/2018 47	Germany	Prospective	22	N/A	16/6	63.7	CE-MRI
Sekido/2020 48	Japan	Retrospective	60	NA	40/20	60	CE-MRI
Park/2014 49	South Korea	Prospective	40	341	26/14	61.1	CE-MRI
Bae/2018 50	South Korea	Retrospective	176	176	56%/44%	66.7	PET/CT
Hotta/2018 51	Japan	Retrospective	59	1200	34/15	66.8	PET/CT
Kim/2019 31	South Korea	Retrospective	166	N/A	94/72	66.7	PET/CT
Ishihara/2018 32	Japan	Prospective	18	34	11/7	62	PET/CT
Kim/2011 52	South Korea	Retrospective	30	N/A	N/A	N/A	PET/CT
Yukimoto/2021 53	Japan	Retrospective	84	168	53/31	62	PET/CT
Crimì/2020 37	Italy	Prospective	36	N/A	25/11	68.5	PET/MRI
Catalano/2021 38	N/A	Retrospective	62	266	37/25	54	PET/MRI

Abbreviations: CE-MRI, contrast-enhanced magnetic resonance imaging; DQCE-MRI, dynamic quantitative contrast-enhanced magnetic resonance imaging; N/A, not available; PET/CT, positron emission tomography/computed tomography; PET/MRI, positron emission tomography/magnetic resonance imaging.

### Comparison of QDCE-MRI Measurements among Metastatic and Nonmetastatic Lymph Nodes in Patients with Rectal Cancer

The mean short-axis diameters in metastatic and nonmetastatic LNs were 6.9 ± 4 and 5.4 ± 0.5, respectively, based on three articles, while the same values for Ktrans were 0.22 ± 0.1 and 0.14 ± 0.1. V _e_ had a mean of 0.34 ± 0.14 for metastatic and 0.34 ± 0.16 for nonmetastatic LNs. The mean values of K _ep_ for metastatic and nonmetastatic LNs were 0.67 ± 0.24 and 0.72 ± 0.37. The differences between metastatic and nonmetastatic LN parameter were significant by using short axis and Ktrans ( *p* < 0.05) ( Table 3 ).

**Table 3:** The diagnostic accuracy of different imaging methods in the included studies

Author	Sensitivity	Specificity	Accuracy	Method	Cutoff
Doyon/2015	NA	NA	83%	CE-MRI	5 mm
Ogawa/2016	72.6%	54.7%	63.7%	CE-MRI	5 mm
Gröne/2018	72%	45.7%	56.7%	CE-MRI	5 mm
Kim/2018	80.6%	87.3%	91.6%	CE-MRI	5.5 mm
Sekido/2020	81.8%	85%	91%	CE-MRI	5 mm
Park/2014	58%	88.4	NA	CE-MRI	5 mm
Armbruster/2018	71%	70%	NA	CE-MRI	5 mm
Bae/2018	90.6%	70.9%	76.3%	PET/CT	SUVmax for small LNs: 1.1 SUVmax for large LNs: 2.1
Hotta/2018	78.6%	95.4%	91.7%	PET/CT	NA
Kim/2019-PET/CT	48.5%	93.9%	N/A	PET/CT	NA
Ishihara/2018	76.5%	100	N/A	PET/CT	SUVmax: 1.6
Kim/2011	61%	83%	70%	PET/CT	Size > 10 mm
Yukimoto/2021	82.4%	93.4%	92.3%	PET/CT	SUVmax: 1.5
Catalano/2021	92%	86%	90%	PET/MRI	NA
Crimì/2020	90%	92%	92%	PET/MRI	NA

Abbreviations: CE-MRI, contrast-enhanced magnetic resonance imaging; LN, lymph node; NA, not available; PET/CT, positron emission tomography/computed tomography; PET/MRI, positron emission tomography/magnetic resonance imaging; SUVmax, maximum standardized uptake value.

### Meta-Analysis of the Diagnostic Accuracy of Contrast-Enhanced MRI in Distinguishing Metastatic from Nonmetastatic Lymph Nodes

**Fig. 2:**
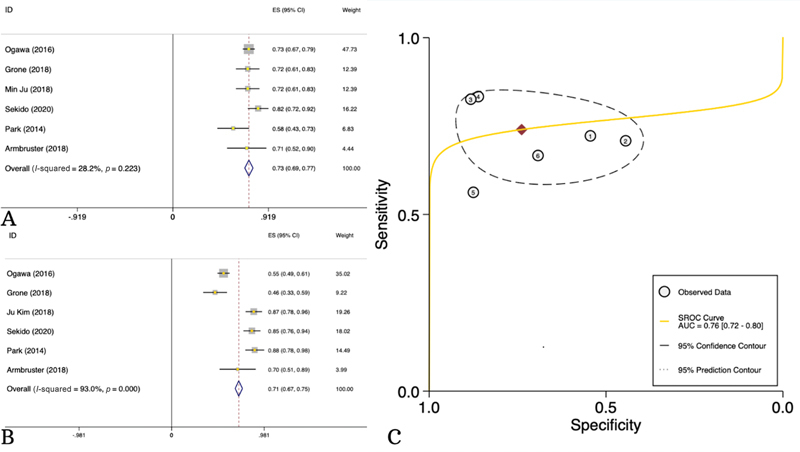
Diagnostic sensitivity ( **A** ) and specificity ( **B** ) and area under the curve (AUC) ( **C** ) of contrast-enhanced magnetic resonance imaging (CE-MRI) in distinguishing metastatic from nonmetastatic lymph nodes.

A total of 6 studies consisting of 530 patients provided data regarding the diagnostic accuracy of CE-MRI in distinguishing metastatic LNs. Almost all of these studies (five) reported the short-axis cutoff point of 5 mm as the optimal cutoff. CE-MRI showed 73% (95% CI: 69–77%, *I* ^2^ = 28%) sensitivity, 71% (95% CI: 67–75%, *I* ^2^ = 93%) specificity, and 79% (95% CI: 76–83%, *I* ^2^ = 93%) accuracy in detecting metastatic LNs among RC patients ( Fig. 2 , Table 3 ).

### Diagnostic Sensitivity and Specificity of QDCE-MRI in Distinguishing Metastatic from Nonmetastatic Lymph Nodes

**Fig. 3:**
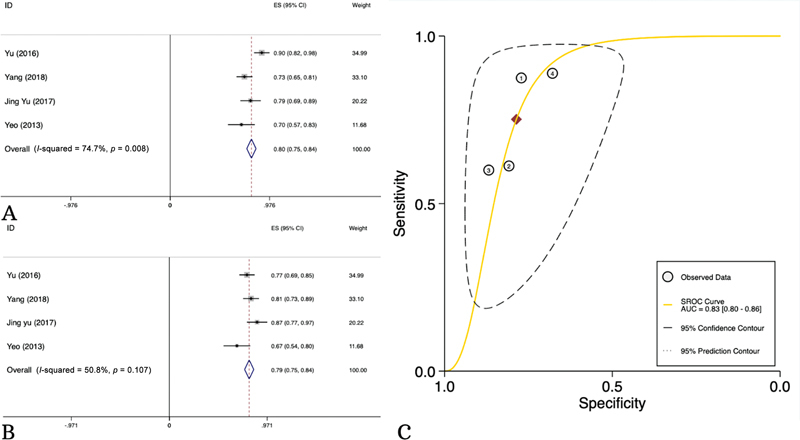
Diagnostic sensitivity ( **A** ) and specificity ( **B** ) and area under the curve (AUC) ( **C** ) of quantitative dynamic contrast-enhanced magnetic resonance imaging (QDCE-MRI) in distinguishing metastatic from nonmetastatic lymph nodes.

Only 4 studies including 290 patients evaluated the accuracy of QDCE-MRI for metastatic LN detection among RC patients. The overall sensitivity, specificity, and accuracy of QDCE-MRI using Ktrans was calculated to be 80% (95% CI: 75–85%, *I* ^2^ = 88%), 79% (95% CI: 75–84%, *I* ^2^ = 51%), and 80% (95% CI: 75–84%, *I* ^2^ = 75%), respectively ( Fig. 3 , Table 4 ).

**Table 4:** QDCE-MRI accuracy in distinguishing metastatic lymph nodes from nonmetastatic ones

Author	K ^trans^				V _e_			
	Sensitivity	Specificity	AUC	Cutoff (min ^−1^ )	Sensitivity	Specificity	AUC	Cutoff
Yu	88.57	77.27	0.897 (0.833–0.962)	0.302	82.86	86.36	0.887 (0.816–0.958)	0.259
Yang	60.5	81.5	0.732 (0.610–0.854)	0.088	N/A	N/A	N/A	N/A
Yu	61.2	87.5	0.788 (0.667–0.881)	> 0.412	N/A	N/A	N/A	N/A
Yeo	88	66.7	0.699	0.09	N/A	N/A	N/A	N/A

Abbreviations: AUC, area under the curve; N/A, not available; QDCE-MRI, quantitative dynamic contrast-enhanced magnetic resonance imaging.

### Diagnostic Sensitivity and Specificity of PET/CT in Distinguishing Metastatic from Nonmetastatic Lymph Nodes

**Fig. 4:**
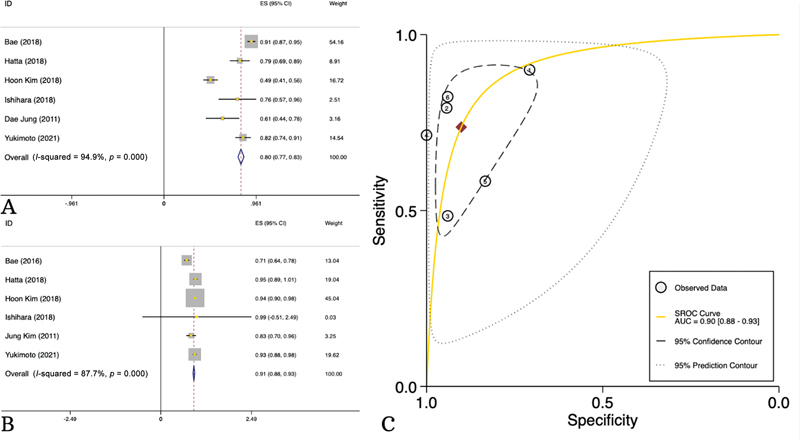
Diagnostic sensitivity ( **A** ) and specificity ( **B** ) and area under the curve (AUC) ( **C** ) of positron emission tomography/computed tomography (PET/CT) in distinguishing metastatic from nonmetastatic lymph nodes.

A total of 6 studies consisting of 533 patients evaluated PET/CT in detecting the metastatic LNs among RC cases. PET/CT showed a sensitivity of 80% (95% CI: 77–83%, *I* ^2^ = 94.9%), specificity of 91% (95% CI: 88–93%, *I* ^2^ = 87.7%), and accuracy of 86% (95% CI: 82–89%, *I* ^2^ = 85.3%) in distinguishing metastatic LNs ( Fig. 4 , Table 4 ).

### Publication Bias

**Fig. 5:**
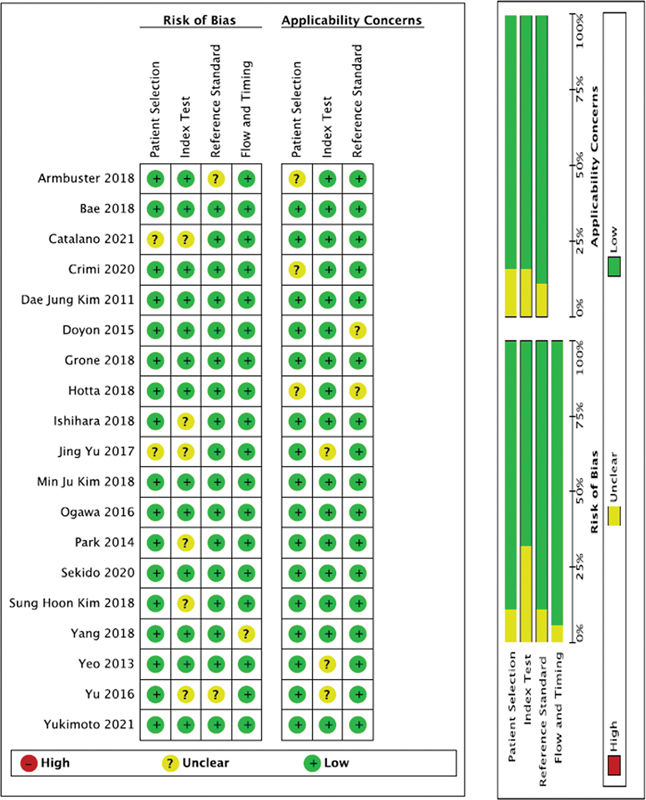
Quality Assessment of Diagnostic Accuracy Studies (QUADAS-2) diagram for publication bias.

QUADAS-2 diagram was created for the included studies. It was showed that there was mostly low or unclear bias ( Fig. 5 ).

### Clinical Utility

**Fig. 6:**
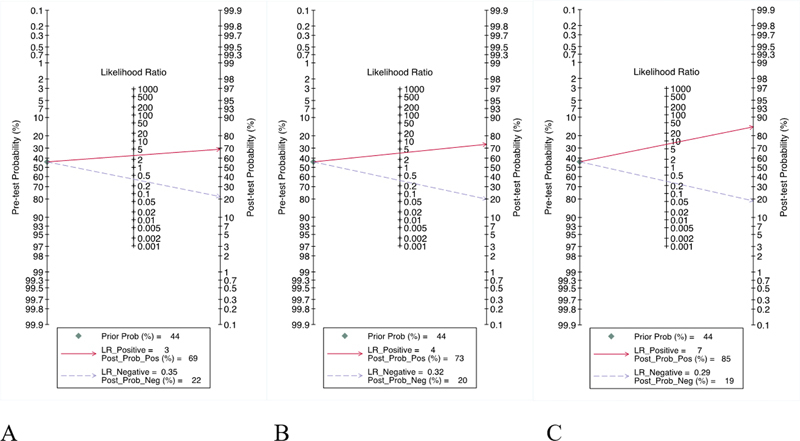
The Fagan nomogram showing the pre- and posttest probability estimation for contrast-enhanced magnetic resonance imaging (CE-MRI) ( **A** ), quantitative dynamic contrast-enhanced MRI (QDCE-MRI) (Ktrans)(B), and positron emission tomography/computed tomography (PET/CT) ( **C** ) in distinguishing metastatic lymph nodes from nonmetastatic ones among rectal cancer patient.

Using CE-MRI, QDCE-MRI, and PET/CT would increase the posttest probability to 69, 73, and 85%, respectively. The PLRs for these three methods were 3, 4, and 7, respectively. This means that in case of a positive CE-MRI, QDCE-MRI, and PET/CT there will be 69, 73, and 85% chance, respectively, to have a metastatic LN and in case of their negative results there will be 22, 20, and 19% chance, respectively, to still have a metastatic LN ( Fig. 6 ).

## Discussion

Our meta-analysis centered on evaluating the efficacy of CE-MRI, QDCE-MRI, and PET/CT in distinguishing metastatic from nonmetastatic LNs in RC patients. Our main findings revealed that QDCE-MRI and PET/CT exhibit acceptable diagnostic sensitivity.

It is recognized that metastatic LNs typically exhibit enlarged short-axis diameters in CE-MRI. 9 Aligning with prior research, our data also displayed elevated short-axis diameters in metastatic LNs. Most of the included studies considered short-axis value of 5 mm as the cutoff value leading to a pooled sensitivity and specificity of 73 and 71%, respectively.

A more accurate imaging method can be QDCE-MRI. It is well-established that vascular formation plays a pivotal role in tumor proliferation, correlating with transmural expansion, local lymphatic dissemination, and distal blood-borne metastasis in colorectal malignancies. 20 Ktrans is a pivotal measure of the rate at which the gadolinium contrast medium transfers from blood plasma to the extracellular extravascular space, providing a quantitative assessment of tissue perfusion and capillary permeability. 21 22 23 Ktrans is highly valued for its ability to indicate the permeability of capillaries within a tissue, which is crucial for assessing tumor angiogenesis and the effectiveness of neoadjuvant therapies. 24 Studies have shown that higher Ktrans values are often associated with increased vascular endothelial growth factor and epidermal growth factor receptor expressions, which are markers of aggressive tumor behavior and angiogenesis. 25 26 27 28 29 30 Our results demonstrated that QDCE-MRI, particularly through the measurement of Ktrans, exhibits a sensitivity of 80% and a specificity of 79% for detecting metastatic LNs.

Another new method for discriminating metastatic LNs is PET/CT scan which recently has shown a potential complementary role through its advantage in assessing the metabolic activity. 31 The pooled sensitivity of PET/CT and QDCE-MRI seemed comparable based on our study. Meanwhile, PET/CT showed a specificity of 91%, which makes it more accurate than QDCE-MRI.

The pooled sensitivity of PET/CT and QDCE-MRI seemed comparable based on our study. Meanwhile, PET/CT showed a specificity of 91%, which makes it more accurate than QDCE-MRI.

The reported sensitivity measures for PET/CT were very heterogenic among the studies ranging from 49 to 91%. This heterogeneity can probably be explained by the difference in the threshold values and the biomarkers used. 31 32 33 The sensitivity of PET/CT can be very low, while showing a very high specificity. This is because FPs can occur due to inflammation or the presence of the venous plexus, making this method not entirely reliable for diagnostics. Consequently, alternative biomarkers like peak standardized uptake values (SUVmax and SUVpeak) and metabolic tumor volume are being used to predict LN involvement. 34 35

When combining size ≥ 12 mm with SUVmax ≥ 1.6, Ishihara et al found a 92.9% accuracy, 88.2% sensitivity, and 84.6% negative predictive value for FDG-PET/CT in detecting metastatic LNs. 32

Meanwhile a significant drawback of PET/CT is its limited spatial and contrast resolution for soft tissues, preventing accurate assessment of mesorectal LNs smaller than 5 mm in diameter, which is below the resolution capability of PET. Furthermore, blooming artifacts resulting from the uptake in primary lesions can mask the uptake in smaller adjacent LNs. These limitations could potentially be addressed by using PET/MRI. 36

The simultaneous detection of FDG uptake and superior soft tissue contrast in PET/MRI makes this method another valuable tool for identifying small, abnormal LNs. We were only able to find two studies evaluating the diagnostic ability of PET/MRI in distinguishing metastatic LNs from nonmetastatic ones. 37 38 Both of these studies showed great sensitivity, specificity, and accuracy. 37 38 Crimì et al conducted a study on the restaging of 36 patients with locally advanced RC following chemoradiotherapy, demonstrating that whole-body FDG-PET/MRI was marginally more accurate than MRI alone in assessing N (92% vs. 86%) stages. Additionally, PET/MRI results led to alterations in treatment plans in 11% of the cases, particularly when hypermetabolic tumor residuals were identified within fibrotic areas. 37

Furthermore, Li et al in their study evaluated the diagnostic precision of PET/CT and MRI in identifying LN metastasis in RC. Their results showed a 89.9% sensitivity and 90.5% specificity for PET/CT when using a SUV value of 2.0 as the diagnostic threshold. 33 Meanwhile, the combined application of PET/CT and diffusion-weighted imaging MRI not only validated their individual efficacy but also showcased a synergistic enhancement in diagnostic accuracy, with the combination reaching an accuracy of 94.4% for detecting metastatic versus nonmetastatic LNs. 33

Therefore, the integration of these modalities, each with its unique strengths in specificity, sensitivity, and the ability to monitor therapeutic outcomes, illustrates the advancing front of diagnostic precision in managing RC.

## Limitation

A notable limitation of our analysis was the inherent heterogeneity across the incorporated studies and the variability in threshold values.

## Conclusion

Our meta-analysis highlights the evolving landscape of imaging modalities for identifying metastatic versus nonmetastatic LNs in RC patients. QDCE-MRI, with its focus on the Ktrans parameter, and PET/CT, assessing metabolic activity, both show substantial diagnostic potential.

QDCE-MRI demonstrates a commendable sensitivity and specificity, but slightly overshadowed by the higher specificity of PET/CT at 91%, despite comparable sensitivities. However, the heterogeneity in PET/CT sensitivity across studies and its high specificity indicate variability that can influence clinical decision-making. Thus, combining these imaging techniques and perhaps newer methods like PET/MRI could enhance diagnostic accuracy, reduce variability, and improve patient management strategies in RC.
